# Stabilization of Comminuted Fractures of the Proximal Humerus with Intramedullary Nails and Angularly Stable Locking Plates—Functional Results before and during the SARS-COVID-19 Pandemics

**DOI:** 10.3390/medicina59030575

**Published:** 2023-03-15

**Authors:** Karol Ratajczak, Grzegorz Szczęsny, Wiesław Tomaszewski

**Affiliations:** 1 Department of Orthopedic Surgery and Traumatology, Medical University, 02-005 Warsaw, Poland; 2 ARS MEDICA Foundation for Medical Education and Promotion of Health, Art and Culture, 04-036 Warsaw, Poland

**Keywords:** humeral fractures, fractures, comminuted, internal fixation, bone plate, intramedullary nailing

## Abstract

*Background and Objectives*: Intramedullary nailing (IMN) and angularly stable plating (ASP) are the most popular techniques for the stabilization of comminuted fractures of the proximal humerus, without either one being obviously superior. The aim of the study was to validate the functional outcomes of both stabilization techniques in the COVID-19 pandemic by comparing them with data obtained just before the pandemic, because the limitations of the COVID-19 pandemic are affecting several aspects of social and medical life—being afraid of the transmission of the infection, patients reduce their exposure to healthcare to absolutely essential emergencies. Moreover, working conditions in the operating theater have also become more restrictive. *Materials and Methods*: Investigations were performed on 112 adult patients with Neer’s three- and four-fragment fractures stabilized with IMN (64) and ASP (48). Treatment effects were validated six months after surgery based on radiographs for evidence of bone union, humeral neck–shaft angle (NSA) and implant placement. Limb function was assessed with the QuickDash and Constant–Murley scores. Data obtained from patients treated in the COVID-19 pandemic were compared with those obtained before the pandemic. *Results*: The healing of all fractures was satisfactory, but complications developed in six cases. Three patients required secondary interventions due to inadequate repositioning: one after IMN and two after ASP. Additionally, one ASP was complicated by the secondary destabilization of a primarily properly stabilized major tubercle, and in two cases by conflict of the protruding implant with the acromion. ASP was noted to provide better functional results during the COVID-19 pandemic according to the Constant–Murley score (*p* = 0.0048; Student’s *t*-test). No significant differences were observed in the pre-COVID-19 pandemic. *Conclusions*: Our results suggest that ASP is more beneficial for the stabilization of comminuted fractures of the proximal humerus during the COVID-19 pandemic.

## 1. Introduction

Fractures of the proximal humerus occur in patients at all ages, but their frequency increases with senescence [1]. In the population 65-years-old and older, this is the third most frequent fracture site [2], accounting for about 6% of all fractures [3,4] and reaching as much as 10% in the eighth decade of life [5]. They are also the third most common site of osteoporotic fractures. Since 1970, the incidence of these fractures has increased threefold [6]. By 2030, their presumed number may even triple due to the aging of the populations in developed countries [7].

Comminuted fractures of the proximal humerus may be complicated by dislocation of the shoulder and disruption of the surrounding soft tissues [8], including the joint capsule, the ligaments that stabilize it, the surrounding muscles and tendons and even neurovascular bundles [9]. Thus, malunion, soft tissue fibrosis and brachial plexus palsy may affect limb function. Moreover, disruption of vasculature may impair blood supply, affecting bone union, thus promoting, in some cases, nonunion and avascular necrosis of the humeral head [10].

An optimal treatment method depends in each case on the type of fracture (the number of bone fragments, their displacement and concomitant shoulder dislocation) and patient-specific factors, such as the patient’s expectations, his physical activity, the acceptance of the proposed treatment’s method and the willingness to cooperate during the treatment and forthcoming rehabilitation. The final outcome also dependents on the patient’s health status, particularly on severe concomitant systemic burdens that make surgical treatment problematic and even dangerous, such as cardiovascular and respiratory failures, uncontrolled diabetes, severe liver or kidney insufficiencies, acute pancreatitis, neoplasms and many others. Nondisplaced or minimally displaced fractures may be successfully managed with conservative treatment. The treatment consists in limb immobilization in Dessault’s sling for an appropriately long period of time, usually three weeks. Plaster casts are nowadays not recommended due to the lack of comfort during the treatment. Immobilization should not exceed three weeks, as longer timelines increase the risk of shoulder contractures.

Displaced fractures and fractures accompanied with dislocations require joint reduction and anatomical, or as much close to anatomical as possible, reduction of bone fragments with their appropriate fixation. However, a growing number of patients prefer surgical treatment even in nondisplaced or only minimally displaced fractures. This usually occurs in young, physically, professionally and socially active patients whose physical activity is high. These patients appreciate the benefits of surgical stabilization that provides restoration of limb’s functionality without the need of long-lasting immobilization, much easier and faster rehabilitation and a considerably lower risk of contractures and muscle atrophy. These factors, however, are not dominant in middle-aged and elderly patients. In this particular group of patients, general health status and severe comorbid diseases limit the possibility of surgical intervention. Moreover, the activity of these patients is considerably lower, which corresponds to resulting lower expectations. Thus, in summary, in older patients, indications for surgical treatment are not so obvious. Under several circumstances, the benefits coming from anatomical bone reduction do not counterbalance the risk of aggravation of severe comorbidities, especially when persistent disease of the musculoskeletal system, i.e., advanced osteoarthritis, does not allow to fully restore the limb’s functionality.

In several studies, no functional differences between operative and conservative treatments have been noticed at the distant observations, proving that the indications for surgical intervention in this particular group of patients are questionable. It is estimated that, according to Neer’s classification, more than 89% of fractures are not displaced, and thus should be treated conservatively [11,12,13]. Still, when they are displaced, operative intervention is justified, as stabilization enables faster rehabilitation, preserving limb function [14,15]. If stabilization is impossible or, for some reason, unsuitable, there is a rationale for replacing the broken elements with artificial ones (hemiarthroplasty or reverse shoulder arthroplasty) [16]. Nevertheless, osteosynthesis remains the preferred surgical method of treatment, as it preserves anatomical structures and provides for better long-term results. A consensus on which types of fractures should be stabilized and which should be replaced is still under debate [17,18].

Nowadays, comminuted fractures are preferably fixed with intramedullary nails (IMNs) or angularly stable plates (ASPs). The former is considered to be more durable, faster and technically simpler, but the second, owing to a wider surgical approach, simplifies repositioning, thus enabling anatomical restoration of the shape of the broken bone [19]. Both have advantages and disadvantages that impact on final results. Neither seems to be superior over another [20]. Obviously, the restoration of anatomical structures improves healing and thus should result in better limb functionality. However, forthcoming enhanced scarring impairs the shoulder’s mobility, requiring intensive rehabilitation. 

The limitations of the COVID-19 pandemic are affecting several aspects of social life. Afraid of transmission of the infection, patients reduce their exposure to healthcare to absolutely essential emergencies. In consequence, access to physiotherapy has decreased rapidly, adversely affecting the final functional outcomes of the surgical treatment. Moreover, several restrictions were also introduced into medical facilities, interfering with patients’ interpersonal contacts with medical staff at the hospital, outpatient clinic and physiotherapy.

The first case of SARS-CoV-2 infection was registered in Poland on 4 March 2020, and the million mark was reached on 1 December. The rapid spread of the infection disrupted several aspects of social life, supported by legislative countermeasures (the first on 2 March 2020) that focused on constraining dissemination of the virus, including lockdowns, closures of educational, cultural and public institutions, and limitations in public transport and trade. Misinformation spreading through social and mass media led to social tensions resulting in the limitation of interpersonal contacts to those absolutely essential. In consequence, contacts with medical facilities declined, as some facilities were temporarily closed. Patients also reduced their contacts with health professionals to an absolute minimum of their own accord. Unfortunately, physiotherapy happened to be among the most affected medical specialties. As a consequence, reduced accessibility to physiotherapy affected the outcomes of orthopedic treatment. 

The sequelae of a SARS-CoV-2 infection should not be ignored. According to the latest discoveries, SARS-CoV-2 complications include avascular bone necrosis (AVN), which, if missed, may lead to negative outcomes and bone collapse [21].

The aim of the study was to validate the functional outcome of stabilization of comminuted fractures of the proximal humerus with IMN and ASP in the COVID-19 pandemic in comparison to those obtained just before the onset of pandemic.

## 2. Materials and Methods

Retrospective investigations were performed on adult patients who underwent stabilizations with IMN or ASP for comminuted (Neer’s three- and four-fragment [22]) fractures of the proximal humerus between 1 March 2020 and 30 June 2021 (COVID-19 group), comparing them with data obtained before the onset of the COVID-19 pandemic, namely, between 1 January 2017 and 30 September 2019 (reference, pre-COVID-19 group). The follow-up period, including postoperative physiotherapy of up to six months, had to be completed before 1 March 2020 (pre-COVID-19 group) and 31 December 2021 (COVID-19 group). 

Ultimately, 112 patients met the above criteria and agreed to participate in the study, including 68 women and 44 men aged 25–86 years (66.7 ± 14.0 years; mean and SD). In the vast majority, the fractures were a consequence of low-energy injuries. Neither age nor sex had any effect on the treatment outcome—they were statistically insignificant.

Intramedullary nailing was performed in 64 cases using CHARFIX2 (ChM, Juchnowiec Kościelny, Poland) or Expert (DePuy Synthes, Oberdorf, Switzerland) nails, and plate fixation in 48 cases using either Philos (DePuy Synthes, Oberdorf, Switzerland) or ChM 5.0 (ChM, Juchnowiec Kościelny, Poland) systems.

The pre-COVID-19 group consisted of 41 patients who had undergone stabilization with IMN and 23 who had ASP stabilization. The COVID-19 group consisted of 17 patients with IMN and 31 with ASP.

On admission, the configuration of the bone fragments was analyzed based on radiographic images in standard anteroposterior (AP) and scapular Y views, and, in selected cases, determined precisely on CT scans. After the surgery, the limbs were immobilized in a Dessault brace for three weeks and subsequently aggressively mobilized under the supervision of an experienced physiotherapist. Each patient received a detailed rehabilitation protocol, which contained not only the information for the physiotherapist, but also the set of exercises enabling the patient to perform physiotherapy at home. Rehabilitation under the supervision of an experienced physiotherapist is, of course, crucial, but properly selected and safe exercises at home allows to speed up and facilitate the recovery process. Patients had the opportunity to contact the investigation team during the study, including an additional follow-up visits and telephone consultations. As far as possible, we also tried to improve patients’ access to physiotherapy, connecting them directly with therapists and staying in touch during the process of rehabilitation.

Postoperative shoulder rehabilitation must be conducted very carefully. Treatment efficacy depends on the extent of the injury, subsequent periarticular tissue scarring, the efforts of the physiotherapist and the patient’s compliance during the rehabilitation process. The main goal is to restore the physiological mobility of the shoulder, muscle strength and motor coordination that are necessary for full limb functional recovery. To satisfy, the patient has to trust the physiotherapist and has to believe that the treatment will be effective. Only then will be willing to cooperate during the treatment. Unfortunately, even under the best circumstances, complete commitment of the rehabilitation team in the physiotherapy, and excellent patient compliance, full restoration of the limb’s functionality to that from before the injury could not be assured.

The fracture leaves behind less or more pronounced disability.

Intensity of the physiotherapy has to be adjusted to the patient’s abilities and expectations, as well as to the stability of the fracture’s fixation. Poor bone quality, usually resulting from severe osteoporosis, high risk of the disturbances of fracture healing and simply the lack of patient compliance may result in the postponement of the decision to begin rehabilitation. In consequence, the risk of shoulder contractures increases. It is important to avoid limb immobilization in a plaster cast or orthosis for more than three weeks.

Physiotherapy should begin as soon as the limb’s immobilization has been removed. The most favorable time is between the fourth and the ninth postoperative week. Initially, rehabilitation includes passive exercises, with passive–active exercises and active isometric, concentric and eccentric exercises added as limb function improves. The process of rehabilitation should be carefully monitored to avoid complications that may develop in cases where rehabilitation starts too early or is too intensive. These complications may include the loss of fracture’s stability, the implant’s loosening or additional, secondary bone fracture, as well as disturbances in the fracture healing.

The postoperative rehabilitation regime includes has three stages:

- Stage 1 (first 3 weeks)
  − Immobilization with no weight-bearing on the operated limb (using a sling).
  − Initiation of pendulum exercises.
  − Assisted passive movement.
  − Avoiding external rotation for the first 6 weeks.
- Stage 2 (3rd–9th week; may be implemented provided that there are no fracture healing abnormalities or secondary fragment displacement on radiographic images)
  − Assisted active exercises (flexion and abduction in the shoulder joint).
  − Careful active movements with limb abduction until pain occurs, with no weight-bearing

at first, then gradually increasing loads after the 6th week.

- Stage 3 (from 9th week)
  − Isotonic, concentric and eccentric exercises.
  − In patients with normal radiographic bone union progression and with a shoulder joint contracture
  − Passive stretching performed by an experienced physiotherapist.

**Figure 1:**
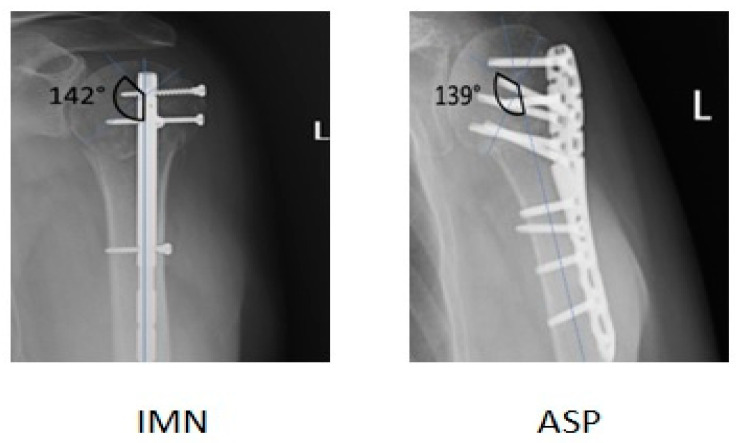
The NSA was measured at the intersection of a line vertical to the anatomic neck and a line parallel to the long axis of the humeral shaft (N = 135° ± 10°) on AP view.

Results were assessed at 6 weeks, 3 months and 6 months postsurgery during follow-up visits. At each follow-up visit, radiographs were obtained in standard AP and Y views. Radiological evaluation was based on bone union, implant placement and the humeral neck–shaft angle (NSA) [23] (Figure 1). Limb function was assessed and assigned to an appropriate group according to the QuickDash (QuickDASH uses 11 items to measure physical function and symptoms in people with any or multiple musculoskeletal disorders of the upper limb) and Constant–Murley scores (a summative scale that provides a global score based on weighted measures of physical impairments in range-of-motion (ROM) and strength along with patient-reported pain and activity limitation (Constant and Murley, 1987)) [24,25,26].

The data are presented as means and standard deviations (SDs). Statistical analysis was performed by a professional statistician using the Student’s *t*-test and Shapiro–Wilk test, as well as multivariate analysis of variance data from specialized software (IBM SPSS Statistics 27, New York, USA). Differences were considered significant at *p* < 0.05.

In this retrospective study, all patients gave their written consent.

The study was approved by a local Ethical Committee (AKBE/77/2022).

## 3. Results

**Figure 2:**
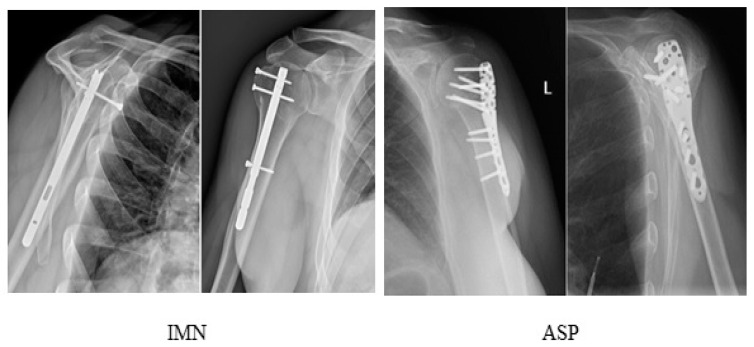
Radiographs of the shoulder at the 6th postoperative month after stabilization with IMN and ASP.

In all patients, follow-up radiographs showed satisfactory healing of bone fractures and, consequently, advanced bone union at the follow-up visit at 6 months postoperatively (Figure 2). Based on the follow-up radiographs, satisfactory results were recorded in 45 out of the 48 patients (93.8%) who had undergone ASP stabilization and in 61 out of the 64 patients (95.3%) stabilized with IMN. Unsatisfactory stabilization was noted in six cases only (5.4%), including three patients from the ASP group (6.3%) and three from the IMN group (4.7%). Of the one hundred and twelve patients analyzed, three (2.7%) required secondary interventions due to inadequate repositioning of the bone fragments, including one (0.9%) in the IMN group and two (1.8%) in the ASP group. Additionally, in one case (0.9%), plate fixation was complicated by secondary destabilization of a primarily properly stabilized major tubercle, and in two cases (1.8%), final results were complicated by a conflict of the implant with the acromion on account of a protruding IMN. There were no cases of infection in the study group (Table 1).

Limb function analyzed according to the QuickDASH and Constant–Murley scales showed slightly better results (lower values) after the IMN compared to the ASP in the pre-COVID-19 group, although the differences were not statistically significant. Nevertheless, the ASP appeared to provide better functional results than the IMN during the COVID-19 pandemic, and these differences were significant according to the Constant–Murley score (*p* = 0.0048; Student’s *t*-test) (Table 2). Good and excellent results were recorded in 73.4% (30/41) and 85.4% (35/41) patients after the IMN and 94.1% (16/17) and 94.1% (16/17) patients after the ASP in pre-COVID-19 group, according to the QuickDash and Constant–Murley scales, respectively. In the COVID-19 group, these values reached 87.0% (20/23) and 78.3% (18/23), and 93.5% (29/31) and 93.5% (29/31), respectively (Table 3). Analysis of the radiographs revealed implant protrusion in two cases after IMN and an inadequate humeral neck–shaft angle in four cases (one after IMN and three after ASP) (Table 1).

## 4. Discussion

Our study showed superior functional results after the ASP compared to IMN during the COVID-19 pandemic at 6 months postoperatively. Interestingly, both methods of fracture stabilization performed by the same operative team revealed no differences in the pre-COVID-19 era, pointing to the factor responsible for this observation. Analyzing our procedures, we came to the conclusion that this observation could be explained by the limited accessibility of physiotherapy during the COVID-19 pandemic, which influenced the final treatment outcomes. Neither stabilization is known to be superior to the other one, as both produce comparable long-term outcomes [27]. Nevertheless, even though the ASP provides for better early functional outcomes, most probably due to more precise restoration of the humeral anatomy [28], this is offset in longer time-frames by faster convalescence and restoration of limb function after IMN [19].

In our study, more ASP stabilizations than IMN procedures were performed during the COVID-19 pandemic. In comparison to the pre-COVID-19 era, the percentage of stabilizations performed with the former method increased from 22.3% to 68.5%. Since every procedure was decided on individually, taking into account the details of a particular case and circumstances, this could be explained by easier stabilization during the COVID-19 pandemic, when procedures had to be performed under a special sanitary (virological) regime. Put simply, ASP, due to its greater ability to restore shoulder anatomy and higher stability of the anastomosis, reduces the risk of secondary interventions due to inadequate stabilization. Moreover, stabilization is possible with minimal use of intraoperative radiographs or even without this guidance, thus reducing the personnel in the operating room (at least less a radiology technician) and solving problems related to the need to decontaminate electronic devices. It is also of value since limited access to postoperative physiotherapy due to COVID-19 restrictions made plate fixations more suitable for limb mobilization under the minimal monitoring of the therapist, including telerehabilitation, or even without it [29,30].

ASP and IMN have dominated the stabilization techniques of the fractures of the proximal humerus, becoming the “gold standard” for these particular types of injuries. Both produce comparable results, as several meta-analyses have concluded that it is impossible to ascertain which one is better [31]. Nevertheless, IMN is associated with lower risk of intraoperative bleeding and rate of infections, shorter surgical time and faster healing of the fracture. It also gives practically identical final therapeutic effects, as the stability, restoration of anatomical structures, range of motion and pain intensity are comparable with those after ASP. Moreover, no significant differences in the number of reoperations, rates of impingement syndrome, delayed union and implant loosening were noted between the two methods [32].

Despite the development of complications in six patients, including three from the ASP group and three from the IMN group, a poor final functional result was recorded in only one case. This could be explained by lower expectations of elderly people (the mean age of the patients in our study was 66 years) compared to younger ones, and a high ability of the shoulder to offset the limitations of its range of motion with scapular mobility [33]. Thus, despite indisputable imperfections in fracture stabilizations, in the vast majority of the patients with complications (five out of six cases), they had no impact on joint mobility in everyday life.

In our study, there were no cases of AVN, presumably due to early surgery, which has been previously shown to significantly decrease the risk of AVN. However, the latest studies showed a close association with SARS-CoV-2 infection [21].

SARS-CoV-2 increases the risk of thromboembolic complications, both due to the formation of intravascular microthrombi [34,35] and in consequence of steroid administration during treatment [36]. Potentially, vascular disturbances may also affect bone union [37]. Furthermore, SARS-CoV-2 infection causes an increase in inflammatory cytokines such as C-X-C motif–chemokine 10 (CXCL10), interferon gamma (IFN-γ), interleukins (ILs) 1 beta, 6, 8 and 17, and tumor necrosis factor alpha (TNF-α), resulting in bone mineral loss, osteonecrosis and chondrolysis [38]. 

## 5. Conclusions

Both ASP and IMN procedures for proximal humerus fractures can achieve satisfactory functional results on long-term follow-up assessment. 

Despite the good results of the surgical treatment in both groups, the final results suggest that, compared to IMN, ASP may be a better choice in the treatment of proximal humerus fractures during the COVID-19 pandemic.

Limited contact of patients with doctors and physiotherapists during the COVID-19 pandemic had a significant impact on the final results of the treatment.

## Figures and Tables

**Table 1:** Radiographic outcomes of stabilizations, including complications (nonunion, implant protrusion and inadequate humeral neck–shaft angle (NSA)).

	Satisfactory	Nonunion	Implant Protrusion	NSA < 125° or >145°
IMN	61/64 (95.3%)	0 (0%)	2 (3.1%)	1 (1.6%)
ASP	45/48 (93.8%)	0 (0%)	0 (0%)	3 (6.2%)

**Table 2:** QuickDash and Constant–Murley scores at 6 months after intramedullary nailing (IMN) and angularly stable plate fixation (ASP), shown as means ± SD in the pre-COVID-19 and COVID-19 groups. (*) *p* = 0.0048; Student’s *t*-test.

Group	Scores	IMN	ASP
Pre-COVID-19	QuickDash	41.7 ± 19.0	37.9 ± 9.5
	Constant–Murley	14.6 ± 5.2	11.9 ± 6.1
COVID-19	QuickDash	43.3 ± 12.5	39.2 ± 11.6
	Constant–Murley	15.8 ± 5.5	12.9 ± 5.0 (*)

**Table 3:** Functional outcomes of IMN and ASP in the pre-COVID-19 and COVID-19 groups according to the QuickDash and Constant–Murley scores.

IMN	Pre-COVID-19 (n = 41)				COVID-19 (n = 23)			
	Excellent	Good	Fair	Poor	Excellent	Good	Fair	Poor
Quick-Dash	12 (29.5%)	18 (43.9%)	10 (24.4%)	1 (2.4%)	2 (8.7%)	18(78.3%)	3 (13.0%)	0
Constant–Murley	9 (22.0%)	26 (63.4%)	6 (14.6%)	0	4(17.4%)	14(60.9%)	5 (21.7%)	0
**ASP**	**Pre-COVID-19 (n = 17)**				**COVID-19 (n = 31)**			
	**Excellent**	**Good**	**Fair**	**Poor**	**Excellent**	**Good**	**Fair**	**Poor**
Quick-Dash	3 (17.7%)	13 (76.5%)	1 (4.4%)	0	5 (16.1%)	24 (77.4%)	2 (6.5%)	0
Constant–Murley	7 (30.4%)	9 (52.9%)	1 (4.4%)	0	14 (45.2%)	15 (48.4%)	2 (6.5%)	0

## Data Availability

The data obtained in this study are presented in the manuscript. Raw data are available on request from the corresponding author.
